# Family Involvement in Learning From Expected Child Deaths: A Qualitative Study of UK Parents

**DOI:** 10.1111/cch.70134

**Published:** 2025-07-07

**Authors:** Joanna Garstang, Anna Pease, Karen Shaw, Jenna Spry, Gayle Routledge, Sara Kenyon

**Affiliations:** ^1^ College of Medicine and Health University of Birmingham Birmingham UK; ^2^ Birmingham Community Healthcare NHS Foundation Trust Birmingham UK; ^3^ Population Health Sciences University of Bristol Bristol UK; ^4^ Bristol Dental School University of Bristol Bristol UK; ^5^ A Child of Mine Stafford UK

**Keywords:** bereaved parents, Child Death Review, communication, learning from deaths

## Abstract

**Background:**

Bereaved parents often have questions about their child's illness and care even when the cause was established prior to death. Child Death Review (CDR) seeks to understand the full reasons for each child's death to help improve care. In the United Kingdom, parents should be informed of CDR, asked for questions or feedback and outcomes shared with them. They should be allocated a keyworker for support with bereavement and CDR. This study aims to explore parents' experiences of CDR following expected child deaths.

**Methods:**

Parents whose children died in England during 2021–2022, in a hospital, hospice or at home with palliative care were recruited through social media, charities and hospitals. Children were aged 1 month to 18 years. Parents had semi‐structured interviews, which were analysed using template thematic analysis.

**Results:**

Parents of 22 children were interviewed. Two integrative themes were generated from analysis: positive and negative CDR experiences. Keyworkers appeared to ensure more positive experiences; these included understanding the purpose of CDR, having answers and reassurance and feeling their CDR involvement could help other families. Negative experiences included confusion around the role of the keyworker, not understanding or being involved in CDR, being left without answers and information from CDR not providing any comfort. Communication and support were the factors driving these experiences. Not all parents wanted to be involved in CDR.

**Conclusion:**

Keyworkers appear to facilitate parental involvement in CDR. Adequate resources and training should be provided for keyworkers to augment learning from child deaths and bereavement support.

## SUMMARY

- Most, but not all, bereaved parents want to be involved in Child Death Review.
- Parents reported positive Child Death Review experiences, including information and reassurance, and feeling that their input may contribute to improvements in care.
- Good communication and support from bereavement keyworkers enable parents to contribute to Child Death Review.
- Bereavement keyworkers provide vital support to grieving families and enable parental involvement in Child Death Review; it is important that this role is appropriately resourced and keyworkers trained in Child Death Review as well as bereavement.
- Although the study was based on the English Child Death Review system, the findings should apply internationally, as Child Death Review occurs in many countries.

## 1 Introduction

In the European Union around 13 000 children die each year before their 18th birthday, equivalent to 3.3 deaths per 1000 live births (European Commission 2024). Approximately one‐third of child deaths are from chronic conditions, malignancy, congenital abnormalities, genetic disease or following a severe acute illness (National Child Mortality Database 2023). The death of a child is one of the most profound and challenging life events that parents ever encounter; bereaved parents suffer more illness (Dias et al. 2018) and have higher mortality (Li et al. 2003) than non‐bereaved parents. Most parents will want and need to understand why their child died (Garstang et al. 2014), as this helps them make sense of loss, an important part of grieving. If the cause of death is unclear or unknown, parents may have more profound grief (Lichtenthal et al. 2013).

When children die from a life‐limiting illness, the diagnosis is usually clearly established before death, and parents will have had conversations with medical staff about treatment options, prognosis and care. Despite this, after a child's death from a life‐limiting illness, parents may have further questions, and studies have described the benefits reported by bereaved parents of follow‐up consultations with paediatricians (Malcolm and Knighting 2021; Hammer et al. 2023). Despite this, many parental bereavement care guidelines often refer only to psychosocial support and not medical follow‐up (Thienprayoon et al. 2018; National Institute for Health and Care Excellence 2019).

Child Death Review (CDR) is the holistic process by which deaths are reviewed in detail aiming to comprehensively understand reasons for each child's death, learning from these to prevent future deaths alongside providing support to families. Although CDR originated with deaths of children from abuse or neglect, it has since expanded to include unexpected deaths, with many countries developing CDR programmes. The American Academy of Paediatrics (AAP) has recently acknowledged the importance of reviewing deaths of children with chronic illness and disability (Batra et al. 2024). All child deaths are subject to CDR in England (HM Government 2018), Wales (Public Health Wales 2024) and Scotland (Healthcare Improvement Scotland 2024). CDR can involve paediatricians, nurses, family doctors, allied healthcare professionals and other agencies such as social care, police, education and public health.

The World Health Organization (WHO) has published CDR guidance for lower and middle income countries as a mechanism to improve paediatric healthcare (World Health Organization 2018). The WHO CDR aims include ‘that families know that their child's life was valued, the death is being taken seriously and health care workers are committed to learning and improving their practice’. However, the WHO guidance does not include information from parents or sharing CDR findings with them. This is potentially a missed opportunity, as parents will have been witness throughout their child's healthcare journey and have unique knowledge of their illness and treatment of which professionals may be unaware (Rosenberg et al. 2016; Khan et al. 2016). For example, a study of CDR for children with complex life‐limiting illness dying at home in Japan described parental mismanagement of equipment failures, but parental narratives did not form part of reviews (Natsume et al. 2022), which limits full understanding of situations and learning from deaths.

There is detailed statutory guidance on the CDR process in England (HM Government 2018) which includes support for bereaved families and investigation of deaths. Multi‐agency CDR meetings (CDRMs) take place some weeks after a child death, attended by professionals who treated or supported the child and family. Only professionals attend these meetings to enable open discussion about medical decisions and treatment. The CDR process should support each family with the appointment of a bereavement keyworker, typically a nurse or bereavement support worker. The keyworker's role is to provide information about processes such as death registrations, social security and funeral directors, helping families access bereavement support and counselling, and to be the main contact around the CDR process; they are not bereavement counsellors. It is expected that keyworkers tell parents about CDR, ask if they have questions or issues for consideration at CDRM, any feedback for clinicians and share with them the outcomes of CDRM. Families should be offered follow‐up appointments with their child's lead clinician. There is detailed guidance available to help parents contribute to the review of infants who die in the perinatal period or are stillborn, as part of the Perinatal Mortality Review Tool (PMRT) (National Perinatal Epidemiology Unit 2023), and for parents following a sudden unexpected child death (Royal College of Pathologists, Royal College of Paediatrics and Child Health 2016). The national guidance gives no further information for keyworkers on how to involve parents or communicate with them about CDR following expected child deaths, such as those from malignancy or life‐limiting disease.

This study aimed to explore parent perspectives of existing CDR practices for expected child deaths. It is part of a co‐design project which examined CDR practice by exploring both parent and health professional experiences and perspectives, which informed the development of a best practice toolkit to support parental involvement in CDR.

This paper reports parents' experiences of CDR involvement; the co‐design work (Garstang et al. 2024) and professional experiences (Shaw et al. 2025) are reported elsewhere.

The specific research question for this study was: What are bereaved parents' experiences of CDR following an expected child death?

## 2 Methods

### 2.1 Study Design, Research Team and Oversight

We used experience‐based co‐design methodology (EBCD) (The Point of Care Foundation 2020), a well‐established process that uses service user and clinicians' experiences collected using qualitative methods to jointly redesign services. The protocol is available at https://doi.org/10.1186/ISRCTN14790455.

Our all‐female research team consisted of JG (researcher and designated doctor for child death), SK (mixed methods maternity researcher), JS (research fellow in palliative care), AP (research fellow in infant death), KS (qualitative researcher in palliative care), AMA (PICU consultant) and GR (bereaved parent) to provide a range of perspectives. We adopted a ‘subtle realist’ approach, acknowledging that phenomena can only ever be observed via the subjective mind of the researcher, but that despite this, such phenomena do exist and can be studied.

We were supported in our study design and delivery by a stakeholder group with representatives from several bereavement support charities; most were already known to the research team from clinical work or previous research projects. We met with the stakeholder group several times during the project. They were involved in all aspects of the study including design, development of interview schedules and topic guides, management, EBCD data analysis and toolkit development (Garstang et al. 2024), and many aspects were altered following their advice. GR (bereaved parent and co‐researcher) led communication with bereaved parents to help address power differences.

### 2.2 Interviews

The study was advertised across England directly to bereaved parents via social media, charity websites and bereavement teams from healthcare organisations. Potential participants were given study information and had informal telephone discussions with JS to check eligibility and answer any questions before agreeing to take part.

Parents could be included in the study if they lived in England, their child had died aged between 1 month and 18 years in 2021 or 2022, in hospital, hospice or at home with palliative care and at least 6 months had elapsed since the death. This period was selected to allow the 2018 CDR statutory guidance to become embedded in hospitals following the COVID pandemic. Parents of infants who had never left hospital since birth, or died in neonatal intensive care units, were excluded as these infants' deaths are reviewed using PMRT.

Semi‐structured interviews were conducted by JS online via Microsoft Teams, face‐to‐face at the family home or by telephone between January 2022 and April 2023. Interviews were audio‐recorded and transcribed. Interviews focused on parents' experiences of CDR and how this could be improved. The topic guide is shown in Table 1.

**TABLE 1:** Interview topic guide.

Broad topic	Details
Background	All about the child, their illness and death
Follow‐up	What contact parents had with healthcare professionals after the death, what was helpful or unhelpful for them.
Questions and concerns about care	Whether parents had questions or concerns about their child's treatment and care, and how these were addressed.
Child Death Review meeting	Parents' knowledge of Child Death Review meetings, their involvement and feedback from them
Best practice and improvement	What parents found most helpful or unhelpful and their suggestions for improvement

### 2.3 Data Analysis

We conducted template thematic analysis, a flexible type of codebook thematic analysis, as described by Brooks et al. (2015) using NVIVO 14 software (NVivo 2023). This followed on from an initial analysis identifying key ‘touchpoints’ for EBCD (Bate and Robert 2006) which meant the whole team were familiar with the transcripts having carefully read these several times. Template thematic analysis was used as a priori themes as well as touchpoints, had been identified during the EBCD process, and this type of analysis allows for the development of themes from the richest data first, in a subset of interviews that captured a cross‐section of the overall experiences from the interviews. We were also able to combine both descriptive and interpretive themes during the analysis to provide insights into the experiences of CDR for bereaved parents that spoke to both their emotional and practical needs.

AP generated an initial coding template based on 10 transcripts; this was then reviewed and refined by JG, by summarizing the content of each code and comparing it between codes and across cases. The remaining transcripts were then coded according to the revised template. The codes were summarized again, renamed to reflect revised content, codes were moved between themes, and relationships between themes were considered. The whole team reviewed the coding template and further refined it, following team discussions.

The codes were then reviewed individually and grouped into themes, considering the mechanisms and pathways that contributed to positive or negative experiences, and a theme map was created.

As part of the initial reading of transcripts, we determined whether parents knew about CDR prior to the research project, their involvement in CDR and satisfaction with their involvement.

### 2.4 Ethics

The project was reviewed by the Health Research Authority and Healthcare Wales on 27 September 2022 reference 22/WM/0172 and was sponsored by Birmingham Community Healthcare NHS Foundation Trust.

## 3 Results

There were 38 bereaved parents who expressed interest in participation; 26 were eligible. There were 21 interviews relating to 22 children: 16 interviews were with mothers, 5 with fathers and 2 with both parents. One audio recording was damaged, so it could not be transcribed, leaving 20 interviews for analysis. There was a wide range of ages, causes of death and ethnicity of children. Details are shown in Table 2.

**TABLE 2:** Details of participating parents and their children.

Category	Description	Number
Age of child	0–4 years	10
	5–11 years	7
	12–17 years	5
Sex of child	Female	11
	Male	11
Ethnicity of child	White	12
	Black or Black British	3
	Other	3
	Not stated	4
Location of death	Home	6
	Hospital	10
	Hospice	6
Cause of death	Chronic illness or disability	15
	Short illness	4
	Cancer	3

### 3.1 Themes and Codes

**FIGURE 1:**
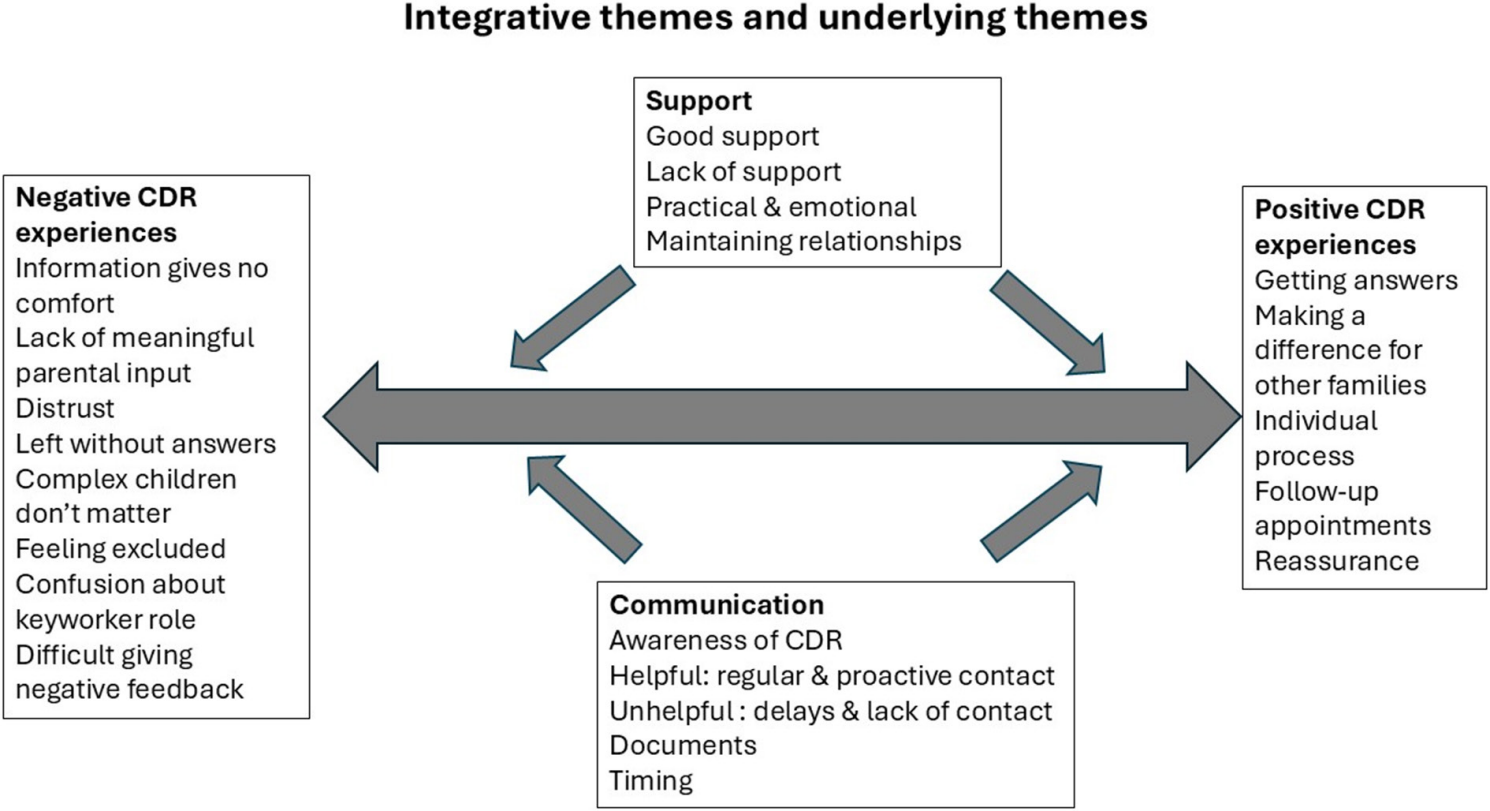
Final codes and themes relating to parents' experiences of Child Death Review.

We identified two integrative themes (themes that include several clusters): positive CDR experiences and negative CDR experiences, with two underlying themes that were the drivers or mechanisms for these experiences: communication and support. The final codes and themes are illustrated in Figure 1. We have included participants' own words to express important ideas in our findings, which we feel allows the authentic voices and perspectives of participants to be clearly heard; these are shown in ‘speech marks’ within paragraphs. Longer quotes are used to provide more detailed and concrete examples to support our interpretations; these are shown as block quotations.

### 3.2 Positive CDR Experiences

Most parents described positive aspects of CDR experiences, explaining how their interactions with professionals shaped these experiences. They highlighted the reassurance and answers provided by professionals along with ongoing follow‐up. Parents' experiences were nuanced in that the same experiences could be interpreted differently by others, showing the importance of individualized approaches to communication and support. Parents' feelings overall about CDR were mixed; many of those reporting positive experiences also reported some negative experiences, but consideration of these experiences overall has enabled us to identify features of best practice. Parents valued the concept of CDR, even if they had not been offered the opportunity to get involved.

I probably would have [wanted to be asked about CDR] … it would have been more a case of posing them questions to see if they've got any learning to come from it. So, for example, when she was a baby, they were talking about doing heart surgery on her, and then they just changed their mind and I never really knew why. (Parent 21)

For many families, getting answers to their questions was ‘really important’ and ‘helpful’, reflecting their need to understand what had happened to their child to help make sense of their loss. After deaths following a short illness parents' questions were medically focused on diagnoses and events leading to the death.

The thing we really wanted from it, which I think we did get, was that we just didn't really know how to describe what happened to her. You know, her surgeon always described it as a stroke, the intensivist always said it was a seizure… And actually what they said was that she had a severe brain stem infection and that was helpful to get that line. (Parent 20)

Parents still had questions when the cause of death was known long before the child died; these were often questions arising after the death or those which parents had wanted answered during the child's life. Questions related to decision making, stopping active treatments and moving to end‐of‐life care. Answers provided reassurance to parents' questions such as ‘did we get everything right?’ about the care and decisions they made for their child. One family who were unaware of CDR were considering legal action to find out if there had been errors in their child's care, feeling they owed this to their child.

She deserves the answers. She was the one that asked us. When she turned 16, she said, ‘Mummy, I know what I want to do with my birthday money.’ I said, ‘What's that?’ She said, ‘I'd like to instruct an expert to see if he did make a mistake.’ (Parent 07)

Some parents appreciated knowing there was a process to learn from deaths ‘it felt really positive that they learn from everything’. It was of comfort to some families that care could be improved to make a difference for others even if the outcome for their child would not have changed. Others were more ambivalent about any change that might come from their feedback as ‘it's left to them to implement that’.

A key part of ensuring positive CDR experiences for families was follow‐up contact with the keyworker. This role was undertaken by hospital bereavement staff, hospice staff or palliative care nurses. Most families had some contact with a keyworker, but the amount of support provided varied considerably; those with less contact had fewer positive experiences. Most families were offered medical follow‐up appointments with their child's consultants; many of the remaining families had wanted this opportunity. These appointments were important to families even if they did not have medical questions; parents often developed close relationships with consultants during their child's illness. These appointments offered comfort and closure as well as answers.

Families needed an individualized approach to CDR; families had different communication and support needs, and not all wanted to participate in CDR. Half of families had questions or gave feedback for the CDRM, but not all found this helpful, in that CDR did not give them answers or answers that gave them no comfort. Some families would have wanted to give feedback and ask questions but were not given the opportunity to do so. Others did not want to give any feedback for the CDRM, mainly because they had no questions about their child's illness, treatment or death.

I just got the letter and I just threw that away … But I would not have written anything on the letter … I didn't have any complaints. I wasn't upset with anybody, so it's fine. (Parent 10)

Parents discussed the importance of having communication that aligned with their preferences for contact. Some valued online meetings like Zoom or Teams, which was considered to offer a ‘more human’ approach, whilst others felt this type of ‘face‐to‐face’ contact was ‘very difficult’ during periods where they felt ‘a complete mess’. For them, email was a preferred option. Thus, the main issue for most parents was being able to choose the manner of communication that best facilitated their involvement.

### 3.3 Negative CDR Experiences

Several parents reported negative aspects of CDR experiences; again, these were nuanced, with many also reporting positive experiences. Negative experiences included the lack of comfort or answers from CDR, challenges contributing to CDR, confusion around keyworkers and distrust of professionals because their child had died. Although some parents were comforted by the information and answers provided to them from CDR, for others, this information did not help with their loss.

… I wasn't really fussed. I don't even want to talk about it now. I cannot bring my children back and so it's about time all the frustration and everything goes away. I'm just leaving it to whatever fate brought to us and we're happy about it. (Parent 09)

Many parents had questions that remained unanswered. Parents who were not involved in CDR had questions relating to their child's illness and treatment ‘I don't really get why she died’. One parent found her follow‐up appointment with the paediatrician too distressing ‘I couldn't speak because it was too soon’, so was not able to ask her questions. Parents whose children had shared care between different services were critical of CDRMs as they lacked important ‘context to answer the question’ which parents could have provided if asked. Several parents were disappointed not to be told outcomes of CDRMs when they had provided information.

It would have been kind of interesting to have had some kind of feedback anyway, if I'd seen the report or something, just to package it, to close it, ‘The report said this and this possibly could have been done, … but it wouldn't have made any difference to the outcome’, because there's always, always doubts … (Parent 14)

Many families found it difficult to provide questions or comments for CDR, mainly due to the lack of guidance and structure in effect being giving a ‘blank page’ to write their feedback; this was an issue for families regardless of support from keyworkers. This difficultly could also relate to the challenge for bereaved parents of processing their emotions.

I was just like oh my God you know how long it's taken me to actually pluck up the courage to call you [keyworker] and now you're saying you want me to put it in an email. So then I tried to write an email and I rewrote the questions and I didn't ask all the questions that I felt I needed to ask because then I got myself all muddled. I feel like I'm quite a literate person to try and ask the questions and I couldn't, you know, I rewrote it about twelve times. (Parent 08)

Some parents explained how they found it ‘sort of felt like a bit of a barrier’ to give potentially negative feedback, not wanting to upset healthcare professionals who may have cared for their child for many years, as the relationship with those professionals was still important to them. Keyworkers could enable the context of parents' feedback to be better understood as they ‘knew what I meant’.

Some parents spoke of how the professionals supporting them knew little about CDR, or the keyworker role, making it difficult for them to contribute to CDR, reflecting the lack of CDR training available. This was further compounded if families were approached about CDR from ‘random’ staff who worked separately from bereavement support teams.

The day that he died, we got given the When a Child Dies booklet but all of it was blank and the Keyworker stuff was blank. I remember asking our Family Support Worker at the time, ‘Will this be you guys?’ She said, ‘Probably not, no, but it won't happen for a few months.’ (Parent 23)

Several parents described how they felt excluded from CDR when meetings or follow‐up appointments did not take place ‘We've not heard from them ever since’. Some felt strongly that similar to meetings during their child's life they should attend CDRMs.

I think the knowledge that a whole bunch of people are meeting up tomorrow morning to talk about her and we're not there… that hurts because she's our daughter … You've got no say and that's hard. (Parent 23)

Several families had concerns about their child's treatment before they died, they then found it more difficult to trust the CDR process or ask questions following the death having previously felt ‘dismissed’ when raising issues. Some parents commented that they felt that as their children had complex life‐limiting conditions, the CDR process would not be thorough as their deaths were seen as inevitable; this reduced their trust in CDR.

I knew my son and I know when he's had enough and he'd had enough, basically, but if it was any other child that didn't have any medical problems, the parents would be in no doubt as to how the child died, wouldn't they? I think it's brushed away almost like it's not important because he was poorly anyway, so it doesn't really matter how he died, but it does to me. (Parent 22)

### 3.4 Communication

Communication was one of the main drivers of parents CDR experiences; most had mixed experiences, describing both helpful and unhelpful communication, as well as the importance of timing and written information. Parents CDR journeys started with being informed about CDR; most were told by healthcare professionals, some as part of planning for their child's death. Others could not recall much about how they heard of CDR. Several parents were unaware of CDR until they participated in this research project.

Parents spoke of the importance of their relationship with their keyworker, experience, communication and understanding of bereaved parents was at the heart of this ‘nothing to do with books or having a degree like a doctor’. Parents valued regular contact with keyworkers who were ‘there whenever I wanted her to be’, were readily accessible, kept parents updated on investigations and managed their expectations on timescales. Parents appreciated keyworkers providing prompt reassurance such as ‘did we do enough for her’ for issues that did not need to wait until the CDRM.

I always felt like I could ask if I needed to talk to her, but she would also check in quite regularly at the beginning … then it got less and less over time. And now, we speak occasionally, usually if I ask, but she messages me on all the important dates. (Parent 06)

Most parents described aspects of unhelpful communication; these frequently concerned lack of follow‐up and delays in discussing results, which increased parents' anxiety. Parents were frustrated by meetings with healthcare professionals who were not present when children deteriorated ‘he wasn't there’ so lacked knowledge to adequately explain events.

So when the postmortem came back, it went to the Child Death Review nurse, and she called and she said, ‘It's in. We can't see you for six weeks’. I was like, ‘Well, don't call’. I'd already waited five months. Just don't call me for another month. Just don't call me, or call me and say, ‘We'll set up a Zoom next week’. (Parent 18)

Parents spoke of their frustration that although keyworkers might have initially contacted them, responsibility for further communication was left to them. They stated it was unrealistic to expect ‘families in crisis’ to ‘take a bit of ownership to get themselves the help’; and that keyworkers should be proactive at keeping in contact. Some parents ‘just didn't ask anything else’, giving up when requests for support did not produce any result. Parents also described the poor communication skills of hospital staff and insensitive actions such as sending key information close to children's birthdays. Parents were sent generic letters from staff they had never met before so did not feel encouraged to make contact. Some spoke of the impact or ‘kick in the teeth’ of seeing the term child death review referring to their child.

Several parents spoke about how they found written documents helpful; after acute illness, parents wanted summaries of children's treatment; all wanted bereavement support and CDR information. For some parents, although mostly helpful, the information was also very distressing ‘like taking your bullet to the chest’ and a ‘hideous book’, such that they only read it several weeks later. Parents spoke of the importance of having the outcome of the CDR ‘much the same as you get reports all the way through every appointment your child has, when they're alive’.

From the interviews, there was no consensus on the right time for keyworkers to tell parents about CDR and ask for their feedback or questions. Some suggested it should be done soon, or parents would forget details; others needed many months to be emotionally ready. One parent expressed confidence that her keyworker ‘will know when I'm ready’ to provide feedback. Others recalled the ‘triggering’ nature of unexpected calls from keyworkers, finding these contacts ‘unhelpful is an understatement’. They suggested keyworkers should give notice of phone calls.

So it was only recently that I've felt a bit more normal if that makes sense? And it's been 13 months now. But then I've also forgotten a lot of things that have happened because I've just blocked it from my memory, so I guess it is important to ask pretty soon after, before you forget everything. But at the same time, you're that numb anyway, you're just a bit like, I don't want to talk about it … . (Parent 15)

### 3.5 Support

Good bereavement support was the foundation for positive experiences, good support consisted of both practical and emotional support and maintaining relationships with bereaved parents. For some parents, the relationship with their keyworker started before the child died as part of palliative care, whereas other families only met their keyworker after the death. This did not impact on the provision of support, the quality of the relationship between parents and keyworker was more important. Good bereavement support involved keyworkers keeping regular contact to offer support, but not making assumptions about what parents wanted. Families took comfort from keeping in contact with healthcare professionals who had cared for their children as ‘they're still part of our lives’.

… our Child Death Review nurse, she didn't try and force herself into coming to see me. I think we probably had three or four phone calls and then I think I said to her, ‘I'd really like you to come round’. (Parent 18)

Parents appreciated practical advice and help from keyworkers; for example, advice around official paperwork, returning equipment and assisting with funeral planning. Several families were supported by hospices after death even if their child had not used their services during life. Hospices were able to support families for much longer than most keyworkers, who usually had to end their support once CDR had been completed.

Many parents spoke of lack of support, or how support could be improved. Frequently, although keyworkers provided information, it was up to parents to arrange their own counselling or psychology support, which was rarely available through the NHS. Parents were frustrated by staff who did not follow‐up their offers of support and left parents the responsibility for ongoing contact when they were only ‘just existing in life’ and ‘you don't know you need help’. Parents wanted a pro‐active ‘I'll come and see you. I'm going to text you’ structured approach for support from keyworkers starting 24–48 h after the death.

We got nothing in terms of like mental health support or anything along those lines. Yeah, it was sort of a case of ‘Oh here's a number for Talk Works if you need it. Off you go.’ (Parent 19)

## 4 Discussion

This paper reports the qualitative interview findings relating to the CDR process for 23 bereaved parents whose children died in hospital, a hospice or at home with palliative care. The keyworker had the most impact on parents' experiences; they facilitated parents' involvement in CDR as well as providing bereavement support. Positive CDR experiences included parents getting answers and reassurance, and parents feeling their CDR involvement could help other families. Negative CDR experiences included the lack of comfort for parents from information, being left without answers, a lack of meaningful parental input to CDR and confusion around the role of the keyworker. Communication and support were the factors driving positive and negative experiences. Keyworkers played a vital role in supporting parents and enabling their involvement in CDR, but to do this, keyworkers needed to understand CDR processes as well as bereavement. Parents valued good, timely communication with keyworkers, assisted by written information. Parents wanted regular contact with keyworkers providing them with emotional and practical support, tailored to their individual needs, helping them access other support services when needed. However, some parents did not want to be involved in CDR as it offered no meaning or benefit to them.

We were able to recruit a wide range of bereaved parents, whose children died from different conditions and with varying involvement in CDR. We learned of good and poor CDR experiences, with parents expressing their preferences for CDR involvement. The project focused on recent CDR experiences after the COVID pandemic, as many healthcare organizations only started to implement CDR after the pandemic. This ensured our findings reflect current practice and challenges, rather than those relating to previous methods of reviewing deaths. Although the study was based within the English CDR system, our findings can be used to support parental involvement in CDR internationally, as many countries have CDR systems, although few have guidance for family involvement. We had a robust process of analysis, including reviewing our overall co‐design project findings with bereaved families and experienced CDR professionals, so these should be reliable and generalizable across families following an expected child death. Although the researchers aimed to set aside their preconceptions, the project was based on improving parents' involvement in the current statutory CDR system, so it did not consider alternative methods of reviewing deaths. Parents who had particularly negative experiences, or who wanted no involvement in CDR, may not have taken part in the project, limiting the learning from these families' experiences or their reasons underlying their preference for non‐participation. The project did not examine cultural contexts of parents' experiences, although the parents who took part were from diverse backgrounds.

There has been limited research on parents' experiences of CDR, as although many countries have CDR, few systems involve parents (Batra et al. 2024; World Health Organization 2018; Natsume et al. 2022). There has been no research on the English keyworker role to date. Our findings that bereaved parents want to understand why their child died as well as practical information and emotional support from professionals are very similar to earlier qualitative research with families after sudden infant death (Garstang et al. 2017). Other studies have reported that after palliative care deaths many parents want follow‐up meetings with paediatricians as well as bereavement support; they have further questions about care (Malcolm and Knighting 2021; Levy et al. 2021), and these meetings can help provide closure from professionals who have been closely involved with families for long periods (Hammer et al. 2023). Our findings concur with a recent systematic review (van Kempen et al. 2022) of follow‐up conversations between bereaved parents' and healthcare professionals. This review reported that follow‐up conversations focused on reviewing children's illness, treatment and terminal events, providing emotional support, and parents giving feedback to improve care and show their gratitude for staff. Professionals often felt undertrained or ill‐prepared for these follow‐up conversations.

Our research adds to growing evidence that bereaved families want more than just emotional support following child death; they seek answers about their child's illness and treatment and wish for opportunities to provide feedback to professionals. Most families, but not all, want to be involved in CDR. Using co‐design with bereaved parents and healthcare professionals, we developed a toolkit to enable parents to participate in CDR (Garstang et al. 2024), which is freely available at https://www.ncmd.info/guidance/parents‐cdr‐toolkit/. This toolkit includes a role description for keyworkers, a structured process for keyworkers to tell parents about CDR, information leaflets, template letters, feedback forms and training resources. This toolkit has yet to be evaluated, and future research should include understanding parents' CDR experiences once the toolkit has been implemented. In addition, further understanding of the impact of CDR on complaints about care and treatment following a child death would be beneficial, along with greater understanding of familial and social consequences.

Keyworkers appear to be important, and without their help, few families are likely to be able to contribute meaningfully to CDR. The keyworker role needs adequate resourcing so that staff have the time and training to provide bereaved families with support to be involved in CDR, as well as emotional support. Without resourcing this role, family involvement in CDR is likely to remain limited, reducing our ability to learn from and potentially prevent child deaths. Most families want consistent, personalized contact from keyworkers, with clear information, practical and emotional support. Keyworkers appear to be important advocates for families, helping them navigate through their bereavement. In this project, parents have identified what support they need from keyworkers, and the CDR toolkit provides a structure for this. The challenge is now to ensure appropriate funding, which will enable equitable access to trained keyworkers for all bereaved families.

## Author Contributions

**Joanna Garstang:** conceptualization, methodology, investigation, funding acquisition, formal analysis, project administration, supervision, writing – review and editing, writing – original draft. **Anna Pease:** conceptualization, methodology, funding acquisition, writing – review and editing, formal analysis. **Karen Shaw:** conceptualization, funding acquisition, writing – review and editing, methodology. **Jenna Spry:** investigation, formal analysis, writing – review and editing. **Gayle Routledge:** conceptualization, funding acquisition, writing – review and editing. **Sara Kenyon:** conceptualization, investigation, funding acquisition, writing – original draft, writing – review and editing, methodology, supervision.

## Ethics Statement

The project was approved by HRA and HCW reference 22/WM/0172 on 27 September 2022; it was sponsored by Birmingham Community Healthcare NHS Trust.

## Conflicts of Interest

All authors have completed the ICMJE uniform disclosure form at http://www.icmje.org/disclosure‐of‐interest/ and declare: This work was funded by NIHR Research for Patient Benefit 203045. J.G. is paid by the National Child Mortality Database as a specialist clinical advisor. J.G. has been paid for developing and delivering educational presentations relating to Child Death for the Primary Care Conference, Stirling Events. J.G. acts as an expert witness for legal cases related to sudden child death; J.G. has received funding from the Lullaby Trust and SUDC‐UK (Sudden Unexplained Death in Childhood) to attend scientific meetings overseas. J.G. is Chair of the Association of Child Death Review Professionals (UK), medical advisor to SUDC‐UK and a member of the Lullaby Trust scientific committee; no other relationships or activities could appear to have influenced the submitted work. S.K. has received funding from NIHR for the following projects: HSDR NIHR151802—Factors influencing the implementation of the Midwifery Continuity of Carer (MCoC) model of care in England, HSDR Listen2Baby study NIHR134306, HTA iHOLDS 17/137/02. She is the labour representative on the British Maternal and Fetal Medicine Society, a member of the HTA MNCH Panel 01/03/2013–31/03/2017, HTA Prioritisation Committee C (Mental health, women and childrens health) 01/03/2017–31/07/2019 and Deputy Chair/member of the NIHR DCAF panel 2018‐ to date. A.P. holds an NIHR Advanced Fellowship NIHR300820 and is Chair of the Lullaby Trust Scientific Committee. G.R. is Chief Executive of A Child of Mine and provides training to professionals about working with bereaved parents in this role; she is trustee of the Children's Cancer and Leukaemia Group. K.S. has received funding from Anthony Nolan Charity, CAR‐T Patient Experience Study; her current post is funded by NIHR BTRU in Precision Cellular Therapeutics. J.S. has received funding from the Association of Child Death Review Professionals to attend their annual conference.

## Data Availability

Data are available upon reasonable request. The data that support the findings of this study are available upon reasonable request to the corresponding author (J.G.).
